# Effect of Red-Beetroot-Supplemented Diet on Gut Microbiota Composition and Metabolite Profile of Weaned Pigs—A Pilot Study

**DOI:** 10.3390/ani13132196

**Published:** 2023-07-04

**Authors:** Opeyemi O. Adekolurejo, Katie McDermott, Henry M. R. Greathead, Helen M. Miller, Alan R. Mackie, Christine Boesch

**Affiliations:** 1 School of Food Science and Nutrition, University of Leeds, Leeds LS2 9JT, UK; o.o.adekolurejo@leeds.ac.uk (O.O.A.); a.r.mackie@leeds.ac.uk (A.R.M.); 2 Faculty of Biological Sciences, University of Leeds, Leeds LS2 9JT, UK; k.mcdermott@leeds.ac.uk (K.M.); h.m.r.greathead@leeds.ac.uk (H.M.R.G.); h.m.miller@leeds.ac.uk (H.M.M.)

**Keywords:** weaned pig, gut microbiota, red beetroot, short chain fatty acids, bile acids

## Abstract

**Simple Summary:**

Weaning causes gut microbiota disruption that results in dysbiosis and post-weaning diarrhea. The recent ban on pharmacological doses of in-feed zinc oxide in the weaned pig diet has made exploration of alternative dietary supplements to improve the post-weaning condition of pigs imperative. Plants (e.g., red beetroot) containing bioactive compounds have shown great potential in this regard, favorably abating gut microbiota dysbiosis and promoting gut metabolite production and health.

**Abstract:**

Red beetroot is a well-recognized and established source of bioactive compounds (e.g., betalains and polyphenols) with anti-inflammatory and antimicrobial properties. It is proposed as a potential alternative to zinc oxide with a focus on gut microbiota modulation and metabolite production. In this study, weaned pigs aged 28 days were fed either a control diet, a diet supplemented with zinc oxide (3000 mg/kg), or 2% and 4% pulverized whole red beetroot (CON, ZNO, RB2, and RB4; respectively) for 14 days. After pigs were euthanized, blood and digesta samples were collected for microbial composition and metabolite analyses. The results showed that the diet supplemented with red beetroot at 2% improved the gut microbial richness relative to other diets but marginally influenced the cecal microbial diversity compared to a zinc-oxide-supplemented diet. A further increase in red beetroot levels (4%—RB4) led to loss in cecal diversity and decreased short chain fatty acids and secondary bile acid concentrations. Also, an increased Proteobacteria abundance, presumably due to increased lactate/lactic-acid-producing bacteria was observed. In summary, red beetroot contains several components conceived to improve the gut microbiota and metabolite output of weaned pigs. Future studies investigating individual components of red beetroot will better elucidate their contributions to gut microbiota modulation and pig health.

## 1. Introduction

Weaning is a stressful phase in pig production characterized by reduced feed intake, poor growth rate, gut microbiota disruption, and diarrhea [1,2]. It is a transitionary phase in the pig life associated with compositional and functional alterations of the gut microbiota, resulting in enteric infections. In pig production, several measures to prevent dire economic losses are currently being explored. Diets provided to weaned pigs have been demonstrated to modulate significant gut microbiota changes leading to increased population of beneficial bacteria species, with remarkable changes observed 10 to 14 days post-weaning [3,4].

Pathogenic colonization of the gut, a leading cause of diarrhea and death in young pigs at weaning, is thus avoidable via dietary modulation of a healthy gut microbial composition [5,6]. Notably, alteration in the composition and diversity of the gut microbiota by the source and level of protein and fiber can increase or deplete gut microbiota metabolite production and corresponding biological responses [7,8,9]. The gut microbiota and metabolite levels in return enhance or inhibit the growth of certain bacteria phyla (e.g., Bacteroidetes, Firmicutes, and Proteobacteria) in the gastrointestinal tract [10,11].

Similarly, in-feed antibiotics and zinc oxide (ZnO) have been reported to reduce piglet mortality during weaning [12,13]. Their capacity to suppress post-weaning diarrhea, alter host-gut microbiota metabolism, and improve feed intake and energy production for growth is well known [14,15,16]. However, despite these advantages, they have been found to destabilize the gut microbial diversity, alter short chain fatty acid (SCFA) levels, and support the emergence of harmful and antibiotic-resistant bacteria species [17,18,19].

As is evident from past literature, in-feed ZnO induces gut microbiota changes characterized by increased coliforms [20,21], reduced anaerobic and lactic acid bacteria [22,23], and reduced commensal bacteria population [24,25]. Consequently, the functional potential of the gut microbiota and its ability to produce health-promoting metabolites (e.g., short chain fatty acids and bile acids) may be compromised.

Additionally, there are concerns about severe environmental pollution from high fecal excretions of zinc [26] linked to in-feed pharmacological doses of zinc oxide coupled with increasing trends of multidrug-resistant *E. coli* [27,28]. This presents a risk to the animal–environment food chain, which necessitated the ban on therapeutic doses of in-feed zinc oxide in weaned pigs across the European Union in June 2022. Thus, it highlights the urgent and continued search for alternatives to in-feed ZnO. There has been an increased research interest in plants containing bioactive compounds; e.g., red beetroot, with health-promoting properties as possible replacements for in-feed ZnO, with emphasis on modulating a healthy gut microbiota and prevention of pathogenic colonization post-weaning [29,30].

Red beetroot (*Beta vulgaris* subsp. *vulgaris conditiva*) contains bioactive compounds such as betalains, polyphenols, inorganic nitrate (NO_3_), fiber, and minerals (e.g., potassium, sodium, phosphorus, calcium, magnesium, copper, iron, zinc, and manganese) [31,32,33]. These bioactives (i.e., betalains, polyphenols, nitrate, and fiber) contribute to the potential prebiotic effect of red beetroot, driving gut microbiota modulation and metabolite production with impacts on host metabolism, physiology, and immune functions [34,35,36].

Red beetroot is one of the top 10 plants with high antioxidant, anti-inflammatory, antimicrobial, anticarcinogenic, and hepatoprotective characteristics [37,38]. Currently, it is being considered as a therapeutic ingredient in the treatment of conditions caused by oxidative stress, inflammation, and metabolic disorders (e.g., hypertension, diabetes, insulin resistance, and kidney dysfunction) [39,40,41]. The health benefits of red beetroot in humans, rodents [42,43], and rainbow trout [44,45] have been widely studied and reported in the literature, but studies using pigs have not been considered, and research demonstrating the potential of red beetroot supplementation of the gut microbiota is still lacking. This study therefore examined the effect of red beetroot on the gut microbiota composition and metabolite output of weaned pigs.

## 2. Materials and Methods

The animal trial was conducted at the National Pig Centre, UK, under an ethical approval granted by the University of Leeds Animal Welfare and Ethical Review Committee (AWERC) under the approval number 070510HM. All husbandry practices were set by the farm in accordance with the Welfare of Farmed Animals (England) Regulations 2007, and all procedures followed the amended Animals (Scientific Procedures) Act 1986. For ethical reasons, the number of piglets per treatment was reduced and determined based on previous studies [46,47,48] that focused on gut microbiota diversity.

The basal diet was provided by Primary Diets, UK, and whole red beetroot powder was purchased from Buy Wholefoods online Ltd. (Ramsgate, UK). Reference bile salts for bile acid quantification were from Sigma-Aldrich (Steinheim, Germany) and Cayman (Cambridge, UK). The mixed short chain fatty acid standard solution containing acetate, propionate, butyrate, valerate, isobutyrate, and isovalerate was from Supelco–Merck Life Science Ltd. (Dorset, UK). All chemicals, solvents, and other reagents used were purchased from Sigma-Aldrich (Germany) and Fischer Scientific (Loughborough, UK) accordingly.

### 2.1. Experimental Animals and Experimental Design

A total of 48 piglets (Large White × Landrace × Duroc) weaned on day 28 (average body weight: 7.58 ± 0.69 kg) were randomly allocated to one of four diets (*n* = 12) while balancing for body weight, sex, and litter origin for a 14-day feeding experiment. The pigs were housed in a temperature-controlled flat deck with open feed troughs and nipple drinkers for easy access to feed and water ad libitum. The experimental diets comprised a basal control diet (CON) and a diet supplemented with 3000 mg/kg zinc oxide (ZNO), both formulated according to the National Research Council (2012) recommendations (Table 1). Red-beetroot-supplemented diets (RB2 and RB4) were obtained by adding 2% (20 g/kg) and 4% (40 g/kg) pulverized whole red beetroot to the basal diet, respectively, and thoroughly mixed with an electric mixer on-site.

During the trial, pigs were weighted individually on days 0, 7, and 14, and feed intake was estimated per diet group for the calculation of the average daily feed intake (ADFI), average daily weight gain (ADG), and feed conversion ratio (FCR). Pig feces on the pen floor was assessed visually by the same personnel and scored on a scale of 1 to 5 (where 1: firm feces; 2: soft feces; 3: mild diarrhea; 4: severe diarrhea; and 5: scour).

### 2.2. Sample Collection

At the end of the experimental period, eight animals per diet (*n* = 8) were euthanized via captive bolt and exsanguination. Blood samples were collected from the jugular vein into heparinized tubes, from which plasma was obtained after centrifugation at 2000× *g* and 4 °C for 10 min. Fecal samples were collected from the rectum into designated tubes. The abdominal cavity was immediately opened, each intestinal segment (duodenum, jejunum, ileum, cecum, and colon) was identified, separately cut, and emptied into a sterile beaker. Digesta from each segment was mixed and aliquoted into sterile 2 mL Eppendorf tubes. All samples were snap frozen in liquid nitrogen prior to storage at −80 °C for gut bacterial composition, short chain fatty acid, and bile acid analyses.

### 2.3. Gut Microbiota Analyses and Bioinformatics

Pig gut microbial composition was examined using digesta samples from the jejunum, ileum, and cecum. Genomic DNA was extracted from (approx. 1.0 g) the digesta samples with a QIAamp Power fecal DNA kit (Qiagen, Hilden, Germany) according to the manufacturer’s instructions. The concentration and purity of the DNA samples were measured spectrophotometrically with a Nano Drop^®^ ND-1000 (Nano Drop Technologies Inc., Dover, DE, USA) using an absorbance ratio at 260/280 nm; those observed were within the range of 1.8–2.0. DNA samples were submitted to the University of Leeds Next Generation Sequencing Facility, St. James Hospital Leeds, UK, for quality screening, 16S rRNA gene library preparation, and sequencing. According to a previous study [49], the V4 hypervariable region of the 16S rRNA gene was amplified in a two-step polymerase chain reaction (PCR) with specific primers (564F and 806R) and an Illumina adaptor overhang. Following the Illumina 16S metagenomics sequencing library preparation protocol, the final libraries were pooled and pair-end sequenced on the Illumina MiSeq platform (Illumina, San Diego, CA, USA).

Sequence reads were processed in Mothur v.1.43.0 with the MiSeq standard operation procedure developed by the Schloss group [50,51]. The chimera-free and unique sequences identified were aligned to the SILVA (v.138) database, and sequences with 97% similarity were clustered into operational taxonomic unit. The “Biome” file generated by Mothur was transferred to the R (v 3.6.2 and 4.0.0) environment for further analyses, including the alpha and beta diversity indices.

The alpha diversity of the gut microbial community was evaluated using the Chao1, Shannon, and Simpson indices, and variables were compared using ANOVA to evaluate the effect of diet, gut location, and their interaction with the lmerTest (linear mixed effects). Differences between gut samples (beta diversity) were determined using a permutational multivariate analysis of variance (PERMANOVA) of the non-phylogenetic distance matrix (Bray–Curtis), which was then visualized on a non-metric multidimensional scale (NMDS) plot. The diet effect on each gut location was computed via a paired comparison of the distance matrices with a pairwise Adonis function (adonis2) in the vegan package (v. 2.6.4).

Differentially (distinct) abundant taxa between gut locations per diet were identified in a two-sided Welch’s *t*-test and Benjamin Hochberg false discovery ratio (FDR) correction in Statistical Analysis of Metagenomics and other Profiles (STAMP) software [52]. Further analysis employed DESeq2 (v. 1.27.32) in R [53] with Wald hypothesis testing for distinct genera in each gut location comparing multiple diet groups. The differences between diets were estimated as the fold change (Log2-fold change) and FDR-corrected *p*-values.

### 2.4. Predicted Functions of Pig Gut Microbiota

To predict the functional pathways mediated by the gut microbiota, OTU abundance and representative sequences processed in Mothur were submitted to Piphillin (https://piphillin.secondgenome.com/ accessed on 12 August 2020). Gene sequences were matched against the Kyoto Encyclopedia of Genes and Genomes (KEGG) database as described in Iwai et al. [54] using USEARCH version 8.0.1623 with the global alignment setting for sequence identification fixed to a 90% cut-off (a level significantly associated with PICRUSt—phylogenetic investigation of communities by reconstruction of unobserved states) [55].

Pathways differentially mediated by diet in the different gut locations were computed with DESeq2 in R using the Wald test and *p*-values adjusted for multiple inter-diet comparisons.

### 2.5. Quantification of Short Chain Fatty Acids (SCFA) and Bile Acids

Short chain fatty acids (acetate, propionate, butyrate, valerate, isobutyrate, and isovalerate) in plasma, jejunum, ileum, cecum, colon digesta, and fecal samples were determined with gas chromatography (Varian 3400; Varian Ltd., Oxford, UK). The method used was as described in Taylor et al. [56] with slight modification. Briefly, a 1.0 g or 1 mL sample was mixed with an equal volume of distilled water in an Eppendorf tube and centrifuged at 12,000× *g* and 4 °C for 10 min. Phosphoric acid (50 µL, 85% *v*/*v*) was added to the supernatant (500 µL) collected alongside 150 µL of caproic acid (150 mM/L) as the internal standard. The mixture was topped up to 1 mL with distilled water and centrifuged at 14,000× *g* for 20 min, after which the supernatant was collected for SCFA analyses. Individual short chain fatty acids in processed samples were quantified using a standard curve obtained from a mixed volatile fatty acid prepared in the concentration range of 0 to 125 mM.

Bile acids in samples were determined as described in Zhang et al. [57]. Briefly, 0.3 g of digesta was mixed with acetonitrile (final conc. 80% *v*/*w*), incubated for 20 min at room temperature, and centrifuged at 15,000× *g* and 4 °C for 20 min. The supernatant collected was passed through Strata-X 33 μm polymer-based solid phase extraction cartridges (Phenomenex, Torrance, CA, USA) after the cartridges had been conditioned with methanol and water. Subsequently, bile acids were eluted in 1.5 mL of methanol, concentrated, dried using a solvent evaporator (SP Genevac EZ-2 Series, Stone Ridge, NY, USA), and then reconstituted in 150 μL of methanol before subjecting them to HPLC-MS (Shimadzu, Kyoto, Japan). Regarding the mobile phase, A and B were a mixture of 5 mM of ammonium acetate in water and methanol, respectively, both acidified with 0.012% formic acid. A mixed standard reference (0–0.1 mM) containing taurohyodeoxycholic acid (THCA), glycohyodeoxycholic acid (GHDCA), taurocholic acid (TCA), glycocholic acid (GCA), taurochenodeoxycholic acid (TCDCA), taurodeoxycholic acid (TDCA), glycochenodeoxycholic acid (GCDCA), glycodeoxycholic acid (GDCA), cholic acid (CA), glycolithocholic acid (GLTHCA), chenodeoxycholic acid (CDCA), deoxycholic acid (DCA), and lithocholic acid was prepared for quantification of bile salts.

### 2.6. Statistical Analyses

SCFA and bile acid concentrations were analyzed in the R environment (v. 4.2.2); zero-inflated data were analyzed using a negative binomial with the square root link function, and the multiple comparison of means was computed using Tukey’s post hoc test with a significance level of *p* < 0.05. Results expressed as the mean and standard error of mean (SEM) are presented in tables. A Spearman correlation analysis was conducted between the SCFA levels, bile acids, and the mean relative abundance of the top 25 genera in each gut location per diet using the “Psych” [58] and “Pheatmap” [59] packages in R (v. 3.31).

## 3. Results

### 3.1. Effect of Diets on Gut Microbial Diversity and Taxonomic Composition

**Figure 1:**
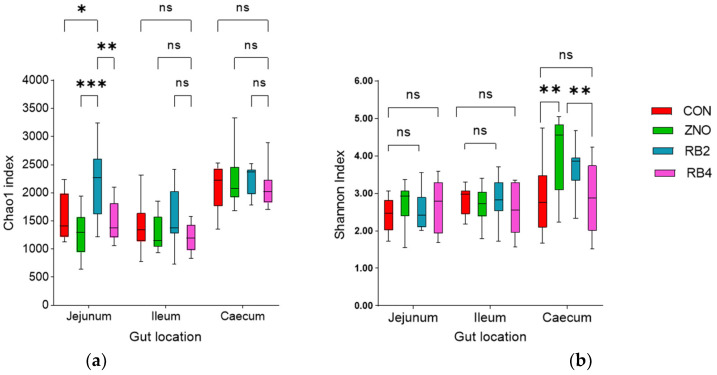
Alpha diversity indices of pig gut microbiota. (**a**) Chao1 index and (**b**) Shannon index showing diet effect on gut species richness and/or diversity. Boxplot represents mean (minimum to maximum) species richness and or evenness from each diet in the gut locations evaluated. A significant difference between the diets linked by a line is indicated by * *p* < 0.05, ** *p* < 0.01, and *** *p* < 0.001 (ns—not significant). CON, ZNO, RB2, and RB4 represent the control diet and diets supplemented with zinc oxide and 2% and 4% red beetroot, respectively.

In the present study, there was no difference in the growth performance or fecal score of the pigs, which is documented in Table S1. However, the diets (*p* = 0.01) significantly influenced the species richness and diversity of the gut microbiota with respect to the gut locations (*p* < 0.001; jejunum, ileum, and cecum) examined. Diet RB2 increased the jejunal species richness (*p* = 0.02) compared to other diets according to the Chao1 index, but the cecal species abundance was comparable for all the diets (Figure 1a). According to the Shannon index of alpha diversity, the gut microbial community was diverse, albeit not influenced by the diets (*p* = 0.07). A pairwise diet comparison showed that the ZNO diet was different from CON and RB4 in the cecum (Figure 1b). No significant species abundance, divergence, or evenness (i.e., dominance) was observed between the diets or in the gut locations for the Simpson index.

**Figure 2:**
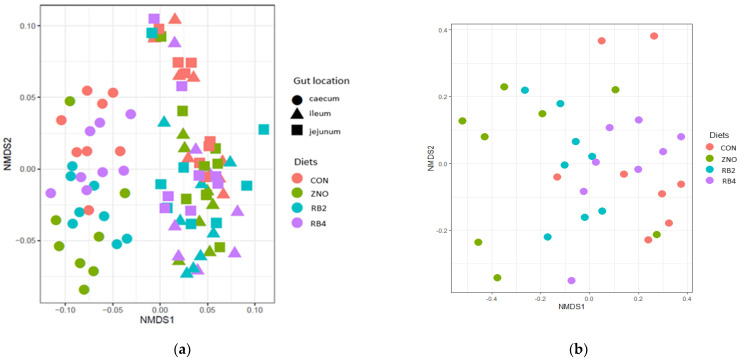
Non-metric multidimensional scaling (NMDS) plots of Bray-Curtis non-phylogenetic distance matrices of gut microbial community of weaned pigs fed different diets: (**a**) distribution of samples by diet and gut location; (**b**) distribution of samples from the cecum. Diets: CON, ZNO, RB2, and RB4 represent the control diet and the diets supplemented with zinc oxide and 2% and 4% red beetroot, respectively.

The beta diversity was as shown in the non-metric multidimensional scaling (NMDS) plots (Figure 2a,b). Samples from the cecum clustered distinctively away from the ileum and jejunum, depicting that the cecum had a significantly different (*p* = 0.013) microbial composition from the ileum and jejunum, while the ileum and jejunum microbial communities were marginally different (*p* = 0.051). A subset analysis of the cecal biome with inter-diet comparisons indicated the CON pigs had more similar species in the cecum than the ZNO (*p* = 0.03) and RB2 (*p* = 0.05) pigs but were related to RB4 pigs (*p* = 0.35). Hence, the ZNO pigs contained more dissimilar species than the RB4 pigs, whereas the RB2 and RB4 pigs were not different (*p* = 0.09).

**Figure 3:**
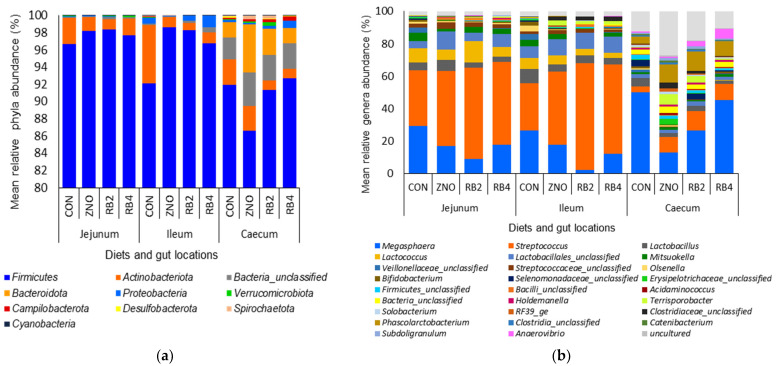
Mean relative abundance of (**a**) phyla and (**b**) genera in pig gut locations with respective diets. CON, ZNO, RB2 and RB4 represent the control diet and the diets supplemented with zinc oxide and 2% and 4% red beetroot, respectively.

In the digesta samples analyzed, 15 phyla and 310 genera were observed with approximately 99% of total sequences (17,573,278) assigned. The mean relative phyla and genera abundance are as presented in Figure 3a,b. The dominant bacteria phyla with mean relative abundance > 1% were Firmicutes, Actinobacteriota, Bacteria unclassified, and Bacteroidota. The mean relative phyla and abundant genera in the gut were compared across the diet groups and are presented in Table 2 and Table S2, respectively. The gut locations mainly influenced (*p* < 0.05) the relative mean phyla distribution with an increase in the cecum compared to other regions examined; however, Firmicutes’ abundance was reduced. Also, an increase in phylum Actinobacteriota and Proteobacteria abundance in the CON and RB4 pigs was observed.

In the top genera (Table S2), *Megasphaera*, *Streptococcus*, *Anaerovibrio*, *Rumminococcaceae*_*unclassified*, *Erysipelotrichaceae*_*unclassified*, *Bacilli_unclassified*, *Terrisporobacter*, and *Clostridiaceae* unclassified abundance were significantly influenced by the diets (*p* < 0.05) and the gut locations (*p* < 0.02) examined. The mean relative abundance of 11 genera (e.g., *Megasphaera*, *Selenomonadaceae_unclassified*, *Phascolarctobacterium*, *Firmicutes*_*unclassified*, *Bacteria*_*unclassified*, *Erysipelotrichaceae*_*unclassified*, *Negativibacillus*, *Anaerovibrio*) functionally recognized as lactate-utilizing bacteria (LUB) were higher (*p* < 0.05) in the cecum but comparable in the jejunum and ileum. Likewise, lower (*p* < 0.05) *Streptococcus*, *Lactococcus*, *Lactobacillales*_*unclassified*, *Streptococcaceae*_*unclassified*, and *Bacilli*_*unclassified* abundance (mostly lactic-acid-producing bacteria—LAB) was observed in the cecum compared to the jejunum and ileum.

### 3.2. Differential Abundant Genera Modulated by the Diets

**Figure 4:**
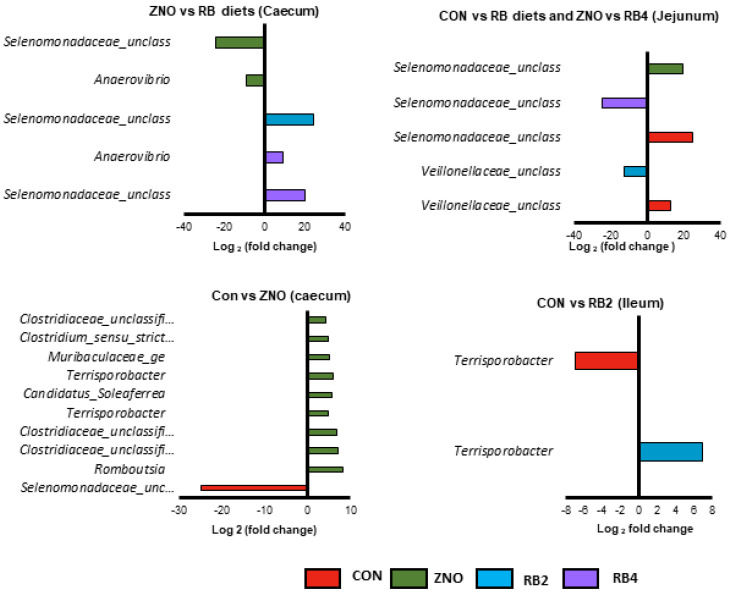
Differentially abundant genera from comparisons between diets in the gut locations with significant (*p* < 0.001) log2-fold changes presented. RB represents red beetroot; CON, ZNO, RB2, and RB4 represent the control diet and the diets supplemented with zinc oxide and 2% and 4% red beetroot, respectively.

Differential abundance at the genus level and distribution per diet were computed using Welch’s *t*-test and FDR-corrected in STAMP. The results indicated an increased (*p* < 0.05) *Megasphaera*, *Selenomonadaceae_unclassified*, and *Veillonellaceae_unclassified* abundance in pigs fed the CON diet along with *Erysipelotrichaceae_unclassified*, *Clostridiaceae_unclassified* and *Rumminococcaceae_unclassified* in the ZNO pigs, while *Bacilli_unclassified* and *Anaerovibrio* increased in the RB2 and RB4 pigs, respectively. Further analyses of each gut location with inter-diet comparisons as shown in Figure 4 presented a decrease in *Veillonellaceae_unclassified* and *Selenomonadaceae_unclassified* abundance in the jejunum of the RB2 and RB4 pigs compared to the CON and ZNO pigs, whereas in the ileum, only the RB2 pigs showed an increased *Terrisporobacter* abundance relative to the CON pigs. The cecum was enriched with nine genera (e.g., *Romboutsia*, *Clostridiaceae_unclassified*, *Terrisporobacter*, *Candidatus_Soleaferrea*, *Muribaculaceae_ge*, and *Clostridium_sensu_stricto_1*) in the ZNO pigs compared to the CON pigs but diminished in genus *Selenomonadaceae_unclassified*. Pigs fed red beetroot diets (RB2 and RB4) had increased cecal *Selenomonadaceae_unclassified* and/or *Anaerovibrio* abundance relative to the ZNO pigs.

### 3.3. Metabolite Profile and Association with Gut Microbial Composition

The short chain fatty acid (SCFA) profile followed the expected pattern of increased levels in the lower gut (cecum and colon), including the fecal samples. Nonetheless, the experimental diets influenced SCFA levels observed in these locations (Table 3). Concomitant with the species richness in the gut locations, SCFA levels increased significantly in the jejunum of RB2 pigs and were reduced in the ileum of ZNO pigs but comparable in the cecum across the diet groups. Overall, the total SCFA levels were reduced significantly (*p* = 0.01) in the RB4 and ZNO pigs, as were most SCFAs (e.g., acetate, propionate, and butyrate).

Similarly, the trend of high jejunal bile acid concentration (approx. 1- to 3-fold) and levels observed in other locations examined was not biologically relevant (Table 4). Pigs fed the RB2 diet had higher (*p* < 0.05) TCA, GCDCA, CA, GLTCA, CDCA, and DCA compared to other diets but had equivalent total and unconjugated bile acids (CA, DCA, CDCA, and LCA) levels with CON pigs. Conversely, the bile acid concentration was reduced in the ZNO (TCDCA, TDCA, GCDCA, GDCA, and CDCA) and RB4 (TCA, GLTCA, DCA, and CA) pigs relative to the CON pigs, which cumulatively ensued lower total and unconjugated bile acid levels.

**Figure 5:**
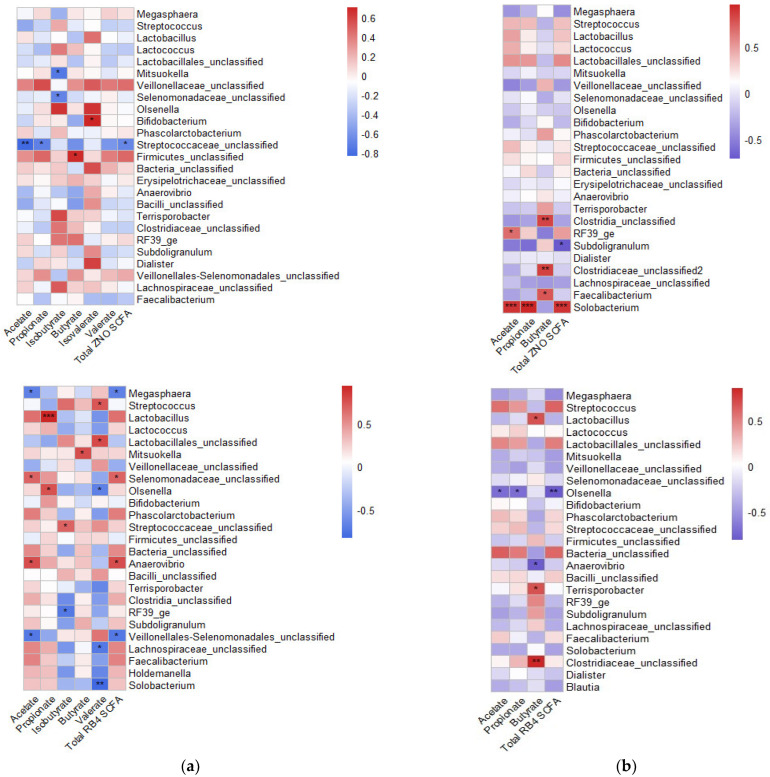
Spearman correlation analyses between top abundant bacteria genera and (**a**) cecal short chain fatty acids and (**b**) ileal short chain fatty acids. Fatty acids omitted were not detected in the corresponding gut locations. The color depth depicts the correlation between the genera and gut metabolite, where a red color denotes a positive correlation and a blue color a negative correlation. The strength of association between the subjects is indicated by the color intensity and *** *p* ≤ 0.001, ** *p* ≤ 0.01, and * *p* ≤ 0.05. CON, ZNO, RB2, and RB4 represent the control diet and the diets supplemented with zinc oxide and 2% and 4% red beetroot, respectively.

Although the total SCFAs in the CON and RB2 pigs was higher, the cecal SCFA levels were comparable across the diet. Acetate and propionate levels correlated significantly with cecal bacteria (e.g., *Firmicutes_unclassified*, *Mitsuokella*, *Megasphaera*, *Streptococcus*, *Streptococcceae_unclassified*, *Anaerovibrio*, *Lactobacillus*, and *Selenomonadaceae_unclassified*) abundance for the CON and RB pigs but were closely associated with the jejunum and ileum (e.g., *Phascolarctobacterium* and *Bacteria_unclassified*) genera abundance in the ZNO and RB4 pigs. Across the gut locations, *Faecalibacterium*, *Blautia*, *Clostridia_unclassified*, *Clostridiaceae_unclassified*, *Dialister*, *Olsenella*, *Selenomonadaceae_unclassified*, *Veillonellaceae_unclassified*, and *Firmicutes_unclassified* were examples of genera that were significantly associated with butyrate in the ZNO and RB4 pigs (Figure 5a,b), most of which were associated with ileal butyrate in the RB2 pigs, although not significant (Figure S1b).

**Figure 6:**
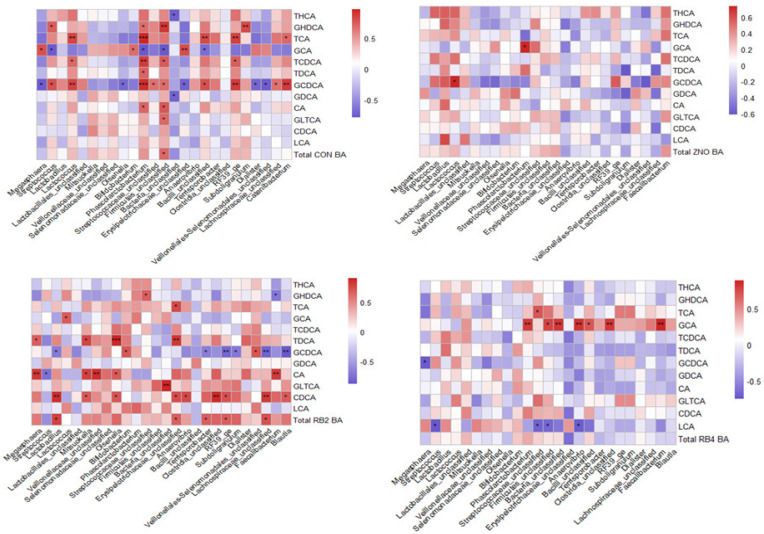
Spearman correlation matrices between jejunal genera abundance and bile acid levels. The bile acid omitted (deoxycholic acid—DCA) was not detected in the jejunum for the pigs and hence is not shown. Correlation depicted by color depth, where a red color denotes a positive and a blue color a negative correlation. The strength of association between the subjects is indicated by the color intensity and *** *p* ≤ 0.001, ** *p* ≤ 0.01, and * *p* ≤ 0.05. CON, ZNO, RB2, and RB4 represent the control and diets supplemented with zinc oxide and 2% and 4% red beetroot, respectively. The bile acids (BA) included taurohyodeoxycholic acid (THCA), glycohyodeoxycholic acid (GHDCA), taurocholic acid (TCA), glycocholic acid (GCA), taurochenodeoxycholic acid (TCDCA), taurodeoxycholic acid (TDCA), glycochenodeoxycholic acid (GCDCA), glycodeoxycholic acid (GDCA), cholic acid (CA), glycolithocholic acid (GLTHCA), chenodeoxycholic acid (CDCA), and lithocholic acid.

Associations between the gut genera abundance and bile acid levels were as presented in Figure S2. In focusing on the unconjugated (CA, CDCA, and LCA) and conjugated (GCA, TCA, GDCA, GCDCA, TCDCA, TDCA, GLTCA, THCA, and GHDCA) bile acids, in the jejunum, the CA, CDCA, and total bile acid levels were strongly associated with most genera in the RB2 pigs, unlike the ZNO and RB4 pigs (Figure 6). Conjugated bile acids were significantly associated with the ilea genera (e.g., *Bacteria_unclassified*, *Selenomonadaceae_unclassified*, and *Veillonellaceae_unclassified*) abundance in the CON and RB pigs; however, most bacteria (e.g., *Lactobacillus*, *Lactococcus*, *Streptococcaceae_unclassified*, *Firmicutes_unclassified*, *Terrisporobacter*, and *RF39_ge*) were associated with unconjugated, conjugated, and total bile acids in the ileum of the ZNO pigs. In the CON, RB2, and RB4 pigs, there was a significant difference between the bacteria genera (*Dialister*, *Streptococcus*, *Lactobacillaes_unclassified*, *Streptococcaceae_unclassified*, *Lactococcus*, and *Selenomonadaceae_unclassified)* and unconjugated bile acids (CA and CDCA) in the cecum (but not in ZNO pigs).

### 3.4. Predicted Gut Microbiota Functions

Compared to the ZNO pigs, the pathways enabling bacteria response and adaptation to environmental changes (e.g., biofilm formation, flagella assembly, and a two-component system) were upregulated in the cecum of pigs on the CON diet. Pigs fed the RB4 diet had pathways influencing lipid metabolism (inositol phosphate, glycerol-phospholipid, fatty acid degradation, chloroalkane, and chloroalkene degradation) enhanced. Aside from these, there were no variations between the diets in the functional pathway predictions from the jejunal and ilea microbiota.

## 4. Discussion

Current reports on the health potential of red beetroot have necessitated evaluations of its probable effect on the gut microbiota and as an alternative to zinc oxide in the weaned pig diet. Red beetroot is a rich source of nutrients, fiber, and bioactive compounds and is recognized for its anti-inflammatory, antioxidant, and antimicrobial properties as well as its prebiotic effect in the gut. Given these benefits, adding red beetroot to a weaned pig diet could promote beneficial microbiota modulation of the gut, thus preventing gut dysbiosis and diarrhea post-weaning.

Diet remains an uncontestable factor shaping and modulating the gut microbiota toward the achievement of gut health and overall wellbeing [60]. In a controlled clinical trial with healthy humans consuming whole cooked red beetroot, Capper et al. [61] showed gut microbiota modulation with reduced Bacteroidetes and increased alpha diversity and short chain fatty acid (SCFA) levels combined with a normal fecal score. The weaning phase in pig production is the focus here due to the attending economic impact, more so due to the recent ban on in-feed ZnO, which has further exacerbated the health implications for weaned pigs.

Studies of the pig gut microbiome have long established that early modulation of the gut microbiota of young pigs is vital to the prevention of post-weaning diarrhea, maturation of the immune system, and improvements in growth performance [2,62,63]. Adaptation of the weaned pig gut to a new diet and achievement of a relatively stable gut microbiota 7 to 10 days post-weaning is essential for early attainment of a richly diverse gut microbial composition and gut health [64].

In the present study, 14-day supplementation of a weaned pig diet with 2% red beetroot (RB2) influenced the alpha diversity, increasing the species richness of the jejunum compared to other diets. However, a comparable number of species was observed in the cecum of all the pigs. Reduced bacteria species in the jejunum [65,66] or ilea digesta [67] of weaned pigs fed a diet with ZnO have been linked to the antimicrobial and growth-promoting ability of in-feed ZnO. According to Bonetti, Tugnoli, Piva, and Grilli [25], in-feed ZnO reduces gut bacteria activity, making more energy available for growth and metabolism.

The cecum had a rich and more diverse bacteria as supposed, whereas the jejunal and ileal microbiota were closely related. This certified the existence of more unique taxa in the cecum compared to the jejunum and the ileum [68]. However, when comparing the RB2 diet with the ZNO diet, the latter clearly modulated a diverse cecal microbiota with more distinct bacteria than the CON and RB4 diets. This was possibly driven by decreased cecal Firmicutes abundance, causing increased relative mean abundance of other phyla (e.g., Bacteroidota). To the best of our knowledge, this is the first report on the supplementation of red beetroot in a weaned pig diet; however, observations of the ZNO diet resonated with previous findings on a pharmacological dose of ZnO in a weaned pig diet [66], while the RB2 diet improved the species richness of the gut.

Meanwhile, increased RB levels did not translate to a diverse cecal microbiota despite increased fiber levels, depicting that the gut microbiota acted differently toward the fiber. In addition, the functions of dietary fiber in the gut are largely determined by its source and physicochemical characteristics (e.g., solubility, viscosity, and fermentability), which subsequently affects the gut microbial composition and metabolite output [69]. Red beetroot contains mainly soluble fiber, which may account for the significant increase in Proteobacteria and reduced SCFAs and secondary bile acids. Bacteria in this phylum tend to increase during weaning stress and in pigs on a diet rich in protein, fat, and fiber, consequently depleting beneficial bacteria like *Lactobacillus*, *Lactococcus*, and *Bifidobacterium* [70].

Firmicutes is consistently the most dominant phylum, accounting for <95% of all phyla observed in the gut [71,72,73]. However, the reduced cecal Firmicutes abundance observed in this current study negated reports of higher populations in the pig cecum [68,74]. Similarly, Actinobacteriota was the second predominant phylum compared to Bacteroidota reported in most studies. While these phyla are important commensals of the gut, the differences in management, experimental diets, sampling age, and location used in these studies may explain the observed disparity [4,75]. However, such gut microbiota alterations were recently attributed to lactate accumulation in the gut.

Wang et al. [76] confirmed gut microbiota variation from lactate accumulation, where Actinobacteria and Proteobacteria replaced the phyla Bacteroidetes and Firmicutes with a concomitant reduction in butyrate and propionate production. Proteobacteria (e.g., *Campylobacter* and *Salmonella* species), which utilize lactate under microaerophilic conditions to produce carbon dioxide and water [77], have predominantly been linked to gut perturbations mostly associated with diarrhea.

The small intestine, which is dominated by lactic acid bacteria—LAB (e.g., *Lactobacilli*, *Lactococcus*, *Streptococcus*, *Bifidobacterium*, etc.), is responsible for lactate production through various biochemical pathways [78]. Lactate prevents the growth of pathogenic organisms by lowering the gut pH value, but increased levels can be harmful and cause alterations in the gut microbiota, toxicity, and pathogenic colonization of the gut. To corroborate this claim, high ilea lactate levels (mM) were observed in the CON (96.76) and RB4 (76.85) pigs, but levels in the ZNO (44.72) and RB2 pigs (48.83) were similar (unpublished).

It is noteworthy that the gut microbiota employs lactate-utilizing bacteria—LUB (from the phylum Firmicutes) with remarkable SCFA-producing ability to avert the detrimental effect of lactate accumulation, thereby stabilizing the gut microbiota [79,80]. Hence, a balance between the LAB and LUB (functional groups) in terms of the production and utilization of lactate is necessary for gut health [77]. Prominent LUB (e.g., *Megasphaera*, *Phascolarctobacterium*, *Negativibacillus*, and *Veillonellaceae*) and LAB (e.g., *Streptococcus*, *Lactococcus*, *Lactobacillales_unclassified*, and *Streptococcaceae_unclassified*) were identified in this study. Importantly, a higher cecal relative LUB abundance but a reduced LAB were observed, while the abundance of both bacteria groups was comparable in the small intestine. These genera were associated with SCFA (acetate, propionate, and butyrate) levels across the gut locations (jejunum, ileum, and cecum) as shown by the correlation matrices.

Though the small intestine is not the major site for microbiota fermentation and SCFA production, significant SCFA levels and correlations with the jejunal and ilea microbiota were observed in the RB diets. The nutritional functions of the jejunum with capacity for energy metabolism and fiber fermentation have been confirmed in many studies, while the gut microbiota metabolite impact the jejunal immune system, barrier function, and cell proliferation [81,82]. In addition, the host immune system is regulated by continuous interaction between the gut microbiota and dietary metabolites; hence, the reduced gut microbiota association with butyrate levels observed in this study may partly be due to host immune responses as well as lactate accumulation in the gut [83]. Moreover, a decline in bacteria sensitivity to metabolite production may have doused a strong correlation between the gut microbiota and butyrate levels in the RB2 pigs, unlike in the ZNO pigs. Overall, inter-individual variability in response to diet as well as variations in gut microbial composition and function cannot be ruled out.

Bile acids have also been linked to host physiology and immunity via gut microbial metabolism. Diet influences the gut microbiota composition and bile acid levels through bile-salt-hydrolyzing bacteria (BSHB) species (e.g., *Clostridium* spp., *Lactobacillus*, *Bifidobacterium*, and *Enterococcus*) that possess inducible genes responsible for the conversion of primary bile acids to secondary bile acids [84,85]. However, interactions between the bile acid and the gut microbiota can be severely impaired in the event of gut dysbiosis at weaning. Song et al. [86] observed that dietary supplementation with CDCA, a natural primary bile acid in animal bile, improved growth performance and reduced diarrheal incidence in weaned pigs.

Generally, the RB2 diet increased individual primary and secondary (CA, CDCA, and DCA) unconjugated bile acid levels compared to the CON diet. DCA (deoxycholic acid) was observed in the colon and feces and hence was not correlated with the gut microbiota abundance. Another study by Tian et al. [87] confirmed a higher and potent antibacterial activity in unconjugated bile acids compared to their other counterpart, and the sensitivity of bile acids to Gram-positive bacteria compared to Gram-negative was also demonstrated. The jejunal bile acid profile was strongly associated with the jejunal microbiota of RB2 pigs (unlike the ZNO and RB4 pigs) but was the same as observed in the ileum for the ZNO pigs. Most bacteria in this region (small intestine) are usually resistant to bile acids, offering protection against pathogenic invasion [88]. Reduced bile acid levels in the gut have been implicated in bacterial overgrowth and inflammation [11]. Conversely, across the diet groups in the cecum, very few genera (e.g., *Streptococcus*, *Lactobacillales_unclassified*, *Lactococcus*, *Selenomonadaceae_unclassified*, and *Erysipelotrichaceae_unclassified*) were involved in bacterial metabolism of the unconjugated bile acids (CA and CDCA). The reasons for the reduced bile acid levels with increased red beetroot is not clear. Usually, a high fat diet increases bile acid discharge, increasing circulating bile acid levels. Alteration of secondary bile acids with dietary fiber and an increased Proteobacteria abundance with the RB4 diet are some possible causes of this trend.

Regarding differentially abundant genera in the pigs, lactate utilizers (e.g., *Veillonellaceae_ unclassified* and *Selenomonadaceae_unclassified*) increased in the jejunum of the CON pigs relative to those fed RB diets, which signified an increased abundance of lactic-acid-producing bacteria (LAB) and a potential for lactate accumulation in the CON pigs. *Terrisporobacter*, an anaerobic Gram-positive bacterium in the family Peptostreptococcaceae, increased in the ileum of the RB2 pigs compared to the CON pigs but was associated with butyrate, GDCA, and GLTCA. Other compositional differences observed in the cecum include increased gut fermenters (e.g., *Romboutsia*, *Muribauculaceae_ge*, *Terrisporobacter*, and *Clostridiaceae_unclassified*) and decreased *Selenomonadaceae_unclassified* in the ZNO pigs compared to the control. Except for increased *Selenomonadaceae_unclassified*, RB2 was not different from ZNO, while RB4 had increased *Anaerovibrio* inclusive.

Generally, *Clostridiaceae_unclassified*, *Rumminococcaceae_unclassified*, and *Erysipelotrichaceae_unclassified* were significantly higher in pigs fed the ZNO diet, which coincided with results of [20] in pigs fed 2425 mg/kg of dietary zinc. The presence of these strict anaerobes demonstrated a rapid transition of the pig gut microbiota from a (milk-based diet) suckling microbiota to a post-weaning (solid-based diet containing complex compounds) microbiota. The preceding genera are linked to bile acid and SCFA production; however, increased *Erysipelotrichaceae* abundance has been implicated in dysbiosis-related disorders of the gut [89] and in mice post-treated with broad spectrum antibiotics (e.g., gentamicin) [90].

Similarly, many studies have confirmed associations between bacteria belonging to this genus and host lipidemic profiles [91,92,93] and cholesterol metabolism [94,95]. This characteristic may be connected to the high systemic and hepatic lipid peroxidation observed in the plasma and liver tissue of pigs in this group (unpublished data) coupled with an increased tendencies for hepatic toxicity and oxidative stress in ZnO-fed pigs. This additionally coincides with the downregulation of pathways facilitating lipid metabolism for pigs fed the ZNO diet compared to those fed the RB4 diet.

Moreover, dietary supplementation with quercetin was reported to inhibit *Erysipelotrichaceae* [96]. Quercetin is a flavonoid (polyphenol) found in fruits and vegetables that is recognized for its health benefits and potential therapeutic effects. Polyphenol and bioactive pigments (betalains) in red beetroot may have been responsible for a decreased abundance of *Erysipelotrichaceae_unclassified* in the RB pigs.

Indeed, red beetroot contains betalains and polyphenols known for effective lipid peroxidation in membrane, thus decreasing oxidative damage [97]. This resonates with observations of upregulated pathways for lipid-metabolism pathways (i.e., inositol and glycerol-phospholipid metabolism) in the cecum of pigs in this group relative to the ZNO pigs. Also, an increased abundance of *Anaerovibrio*, a strictly lipolytic bacteria known for the hydrolysis of triglycerides to fatty acids, in pigs fed a red-beetroot-supplemented diet further confirmed these inferences. Overall, the predicted functional profile from the microbiota of each gut location (jejunum and ileum) did not differ from each diet; aside from what has been earlier described (the cecal microbiota of the CON pigs’ enhanced response to bacteria adaptation to environmental changes).

This study showed the benefit of red beetroot supplementation of the diet of weaned pigs. The ability to modulate the metabolism and function of the gut microbiota demonstrates its potential as an alternative to therapeutic doses of in-feed ZnO to prevent gut dysbiosis and diarrhea that ensues post-weaning. The use of whole red beetroot represents a cost-effective way to provide bioactive compounds (e.g., betalains, nitrate, polyphenols, and fiber) to the pigs at a critical stage in their development. Hence, the effect observed can only be inferred. Future studies utilizing individual components could improve the understanding of red beetroot functionality in modulating the gut microbiota and pig health.

## 5. Conclusions

Diet remains a viable strategy to modulate the gut microbiota of weaned pigs, and red beetroot supplementation provides an avenue to explore its bioactives for pig gut health. In this study, a weaned pig diet supplemented with red beetroot at 2% increased the species richness of the gut microbiota. However, inclinations of lactate accumulation were observed with an increase in RB to 4% (RB4), which was characterized by potential decline in butyrate and propionate and an increased Proteobacteria abundance. The jejunum and ileum microbial compositions were similar across the diet groups, but the cecum was diverse with the ZNO diet relative to the RB2 diet, while the CON and RB4 diets were comparable. The RB2 diet also increased the gut microbiota metabolite (SCFAs and unconjugated bile acids) production in the jejunum and ileum, depicting fore gut fiber fermentation, but butyrate levels were not significantly associated with the gut microbiota as observed in the ZNO and RB4 pigs. The functional pathway predictions from cecal microbiota were closely associated with the distinct bacteria present in the cecum of the pigs across the diets. Altogether, red beetroot has the potential to modulate the gut microbiota of weaned pigs with increased species richness and enhanced lipid metabolism and metabolite production. Future work focused on purified red beetroot components and dosage in weaned pigs is warranted.

## Figures and Tables

**Table 1:** Composition of experimental diets and results for analyzed nutrients.

Ingredients (%)	Control (CON)	Diet with ZnO (ZNO)	2% Red-Beetroot-Supplemented Diet (RB2)	4% Red-Beetroot-Supplemented Diet (RB4)
Barley	15.00	15.00	14.70	14.40
Wheat	28.17	28.17	27.51	26.95
Micronized maize bulk	2.50	2.50	2.45	2.40
Micronized oats	5.00	5.00	4.90	4.80
Fishmeal bulk	6.00	6.00	5.88	5.76
Soya hypro	18.16	18.16	17.80	17.43
Full fat soybean	2.50	2.50	2.45	2.40
Pig weaner premix	0.50	0.50	0.49	0.48
Whey powder bulk	13.89	13.89	13.61	13.33
Potato protein	1.60	1.60	1.57	1.54
Sugar/sucrose	0.63	0.63	0.61	0.60
L-Lysine HCl	0.28	0.28	0.28	0.27
DL-Methionine	0.19	0.19	0.19	0.19
L-Threonine	0.15	0.15	0.15	0.15
L-Tryptophan	0.02	0.02	0.02	0.02
L-Valine	0.04	0.04	0.04	0.04
Vitamin E	0.04	0.04	0.04	0.04
Pan-tek robust	0.02	0.02	0.02	0.02
Sucram	0.01	0.01	0.01	0.01
Benzoic acid	0.50	0.50	0.49	0.48
Pigzin (zinc oxide)	0.00	0.31	0.00	0.00
Di-calcium phosphate	1.13	1.13	1.11	1.08
Sodium carbonate	0.05	0.05	0.05	0.05
* Sipernat 50	0.31	0.00	0.30	0.30
Red beetroot	0.00	0.00	2.00	4.00
Soya oil	3.40	3.40	3.33	3.26
Total (%)	100	100	100	100
Dry matter (%)	89.93	89.65	89.47	89.01
**Analyzed nutrient**				
Ash (%)	6.80	7.50	6.70	6.60
Ether extract (%)	6.73	6.99	6.62	5.92
Crude protein (%)	21.30	21.30	20.70	20.40
Crude fibre (%)	1.90	1.50	1.80	2.20
Zinc (mg/kg)	422.00	2252.00	193.00	187.00

* An inert ingredient made from silica added as a filler with respect to the zinc-oxide-containing diet.

**Table 2:** Comparative analyses of mean relative phyla abundance between diet and gut locations.

Phylum	Diets				Gut Locations			^c^ SEM	*p*-Value		
	CON	ZNO	RB2	RB4	Jejunum	Ileum	Cecum		^d^ L	^e^ D	^f^ L × D
Firmicutes	93.58 ^a^	94.48 ^a^	96.01 ^a^	95.72 ^a^	97.74 ^a^	96.46 ^a^	90.64 ^b^	0.617	0.000	0.312	0.062
Actinobacteriota	4.25 ^a^	1.88 ^ab^	1.06 ^b^	1.42 ^b^	1.93 ^a^	2.51 ^a^	2.02 ^a^	0.347	0.733	0.004	0.224
Bacteria unclassified	0.95 ^a^	1.37 ^a^	1.13 ^a^	1.26 ^a^	0.17 ^b^	0.28 ^b^	3.09 ^a^	0.206	0.000	0.796	0.800
Bacteroidota	0.61 ^a^	1.86 ^a^	1.03 ^a^	0.59 ^a^	0.003 ^b^	0.005 ^b^	3.06 ^a^	0.248	0.000	0.067	0.029
Proteobacteria	0.31 ^ab^	0.11 ^b^	0.31 ^ab^	0.71 ^a^	0.03 ^b^	0.66 ^a^	0.39 ^a^	0.068	0.000	0.005	0.126
Verrucomicrobiota	0.09 ^a^	0.06 ^a^	0.18 ^a^	0.08 ^a^	0.11 ^a^	0.04 ^a^	0.16 ^a^	0.026	0.187	0.389	0.132
Campilobacterota	0.09 ^a^	0.08 ^a^	0.13 ^a^	0.16 ^a^	0.01 ^b^	0.01 ^b^	0.32 ^a^	0.041	0.002	0.916	0.921
Desulfobacterota	0.07 ^a^	0.01 ^a^	0.03 ^a^	0.04 ^a^	0.004 ^b^	0.001 ^b^	0.11 ^a^	0.010	0.000	0.230	0.213
Spirochaetota	0.05 ^a^	0.13 ^a^	0.11 ^a^	0.01 ^a^	0.001 ^b^	0.03 ^b^	0.19 ^a^	0.026	0.003	0.259	0.058

Data represent the mean phyla abundance in each gut location with the different experimental diets; different superscripts (^a,b^) between diet groups and gut location indicate significant differences at *p* < 0.05. ^c^ Standard error of the group mean; ^e^ *p*-value for gut location; ^d^ *p*-value for diet; ^f^ *p*-value for interaction between the gut location and diet. CON, ZNO, RB2 and RB4 represent the control diet and the diets supplemented with zinc oxide and 2% and 4% red beetroot, respectively.

**Table 3:** Short chain fatty acid (mM) profile of diet groups and locations evaluated.

SFCA	Diets					*p*-Value		
	CON	ZNO	RB2	RB4	SEM	* L	^#^ D	^+^ L × D
Acetate	69.14 ^a^	54.76 ^b^	63.81 ^ab^	56.64 ^b^	3.32	<0.01	<0.05	>0.05
Propionate	23.83 ^a^	17.04 ^d^	20.34 ^b^	19.16 ^c^	1.42	<0.05	<0.01	<0.01
Isobutyrate	2.59 ^a^	1.60 ^b^	1.79 ^b^	0.86 ^c^	0.36	<0.02	<0.02	<0.02
Butyrate	9.48 ^a^	6.76 ^b^	8.08 ^ab^	7.09 ^b^	0.61	<0.05	<0.05	>0.05
Isovalerate	1.11 ^a^	0.71 ^b^	0.67 ^b^	0.44 ^c^	0.14	<0.02	<0.02	<0.05
Valerate	1.55 ^a^	0.82 ^b^	0.58 ^c^	0.63 ^c^	0.22	<0.02	<0.02	<0.05
Location								
^1^ Plasma	1.99	1.87	2.31	1.61	0.15	<0.01	>0.05	<0.05
^1^ Jejunum	7.40 ^b^	7.88 ^b^	11.28 ^a^	7.08 ^b^	0.97	<0.01	<0.05	<0.05
^1^ Ileum	9.21 ^a^	7.91 ^b^	9.82 ^a^	8.45 ^a^	0.42	<0.01	<0.05	<0.05
Cecum	24.91	22.36	23.71	22.12	0.65	<0.05	>0.05	<0.05
Colon	24.57 ^a^	19.25 ^b^	17.32 ^b^	22.29 ^ab^	1.61	<0.05	<0.05	<0.05
Feces	39.61 ^a^	22.43 ^c^	30.84 ^b^	23.27 ^c^	4.00	<0.05	<0.05	<0.05
Total SCFA	107.70 ^a^	81.69 ^b^	95.28 ^a^	84.82 ^b^	5.876	<0.05	<0.05	<0.05

Data represent the mean SCFA for each diet group and gut location; different superscripts across the rows indicate significant differences at *p* < 0.05. CON, ZNO, RB2, and RB4 represent the control diet and diets supplemented with zinc oxide and 2% and 4% red beetroot, respectively. ^1^ SCFA levels in these locations were significantly different from levels in the cecum; * *p*-value for effect of location on SCFA levels; ^#^ *p*-value for significant effect of diets; ^+^ *p*-value interaction between location and diet.

**Table 4:** Bile acid profile (nmol/g digesta/feces) from diet groups and locations evaluated.

Bile Acids	Diets					*p*-Value		
	CON	ZNO	RB2	RB4	SEM	* L	^#^ D	^+^ L × D
THCA	40.74 ^a^	27.29 ^b^	16.88 ^c^	26.86 ^b^	4.90	<0.01	<0.01	0.75
GHDCA	33.78 ^b^	46.08 ^a^	25.76 ^c^	33.45 ^b^	4.20	<0.01	0.05	0.62
TCA	1.50 ^b^	1.56 ^b^	1.96 ^a^	1.30 ^b^	0.14	<0.01	<0.01	<0.01
GCA	0.40 ^b^	0.47 ^b^	0.45 ^b^	1.37 ^a^	0.23	>0.05	0.01	>0.05
TCDCA	21.90 ^a^	8.89 ^c^	8.30 ^c^	15.09 ^b^	3.18	<0.01	<0.01	<0.01
TDCA	5.11 ^a^	3.42 ^b^	4.07 ^ab^	3.90 ^b^	0.36	0.02	0.05	0.93
GCDCA	15.42 ^c^	13.88 ^c^	35.91 ^a^	25.40 ^b^	5.10	<0.01	<0.01	0.25
GDCA	3.57 ^b^	4.91 ^a^	2.95 ^c^	3.26 ^b^	0.43	<0.01	<0.01	0.05
GLTCA	2.11 ^ab^	1.99 ^b^	3.60 ^a^	1.96 ^b^	0.40	<0.05	<0.01	0.18
CA	3.80 ^b^	2.59 ^c^	4.23 ^a^	1.63 ^d^	0.59	<0.01	<0.01	<0.01
CDCA	121.16 ^b^	72.28 ^c^	147.09 ^a^	74.32 ^c^	18.34	<0.01	<0.01	<0.01
DCA	0.10 ^b^	0.14 ^b^	0.31 ^a^	0.12 ^b^	0.05	<0.02	<0.02	0.20
LCA	134.25 ^a^	108.90 ^b^	118.20 ^b^	107.66 ^b^	6.13	<0.01	0.05	0.42
Location								
^1^ Jejunum	138.20 ^b^	135.03 ^b^	210.83 ^a^	128.68 ^c^	19.32	<0.01	<0.05	<0.05
Ileum	95.20 ^a^	40.21 ^c^	29.11 ^d^	50.09 ^b^	14.40	<0.01	<0.01	<0.05
Cecum	23.95 ^a^	17.37 ^b^	15.65 ^b^	12.84 ^c^	2.35	<0.01	<0.05	<0.05
Colon	59.88 ^a^	28.45 ^c^	46.40 ^b^	21.10 ^d^	8.77	<0.01	<0.05	<0.05
Feces	66.59 ^c^	71.33 ^b^	67.72 ^c^	83.62 ^a^	3.90	<0.01	<0.01	<0.05
Total unconjugated	259.30 ^a^	183.91 ^b^	269.83 ^a^	183.74 ^b^	23.41	<0.05	<0.05	<0.05
Total conjugated	124.52 ^a^	108.48 ^b^	99.88 ^b^	112.60 ^b^	5.12	<0.05	<0.05	<0.05
Total bile acids	**383.82 ^a^**	**292.39 ^b^**	**369.71 ^a^**	**296.33 ^b^**	**23.98**	<0.001	<0.05	<0.01

Data represent the mean bile acid levels for each diet and location; significant differences are indicated by superscripts across the table for the diet groups. CON, ZNO, RB2 and RB4 represent the control diet and the diets supplemented with zinc oxide and 2% and 4% red beetroot, respectively. ^1^ Jejunal bile acid levels were higher than in other locations examined. * *p*–value for effect of location; ^#^ *p*-value for significant effect of diets; **^+^** *p*-value interaction between location and diet. The bile acids included taurohyodeoxycholic acid (THCA), glycohyodeoxycholic acid (GHDCA), taurocholic acid (TCA), glycocholic acid (GCA), taurochenodeoxycholic acid (TCDCA), taurodeoxycholic acid (TDCA), glycochenodeoxycholic acid (GCDCA), glycodeoxycholic acid (GDCA), cholic acid (CA), glycolithocholic acid (GLTHCA), chenodeoxycholic acid (CDCA), deoxycholic acid (DCA), and lithocholic acid.

## Data Availability

The 16S rRNA sequences (fastq files) generated in this study were deposited in the NCBI Sequence Read Archive (BioProject ID: PRJNA798387). All data analyzed are described in the manuscript, and other statistical outputs are available in the Supplementary Materials.

## Supplementary Materials

The following supporting information can be downloaded at: https://www.mdpi.com/article/10.3390/ani13132196/s1, Table S1: Growth performance and feed intake of weaned pigs. Table S2: Comparison of mean relative abundance of top bacteria genera between diet groups and gut location. Figure S1: Spearman correlation matrices between top abundant bacteria genera and short chain fatty acids in the (a) jejunum and (b) ileum. Figure S2: Spearman correlation matrices between top abundant bacteria genera and bile acid levels in the (a) ileum and (b) cecum.
