# Supplementary material for: Effect of Red-Beetroot-Supplemented Diet on Gut Microbiota Composition and Metabolite Profile of Weaned Pigs—A Pilot Study
Source: Animals (Basel). 2023 Jul 4;13(13):2196. doi: 10.3390/ani13132196 (PMC10339942; doi:10.3390/ani13132196)
Supplement: Supplementary file 1 [file animals-13-02196-s001.zip › animals-2430488-supplementary.pdf]

Supplementary tables and figures

Effect of Red-Beetroot-Supplemented Diet on Gut Microbiota Composition and Metabolite Profile of Weaned Pigs—A Pilot Study

Opeyemi O. Adekolurejo [1,2], Katie McDermott [2], Henry M. R. Greathead [2], Helen M. Miller [2], Alan R. Mackie [1] and Christine Boesch [1,*]

[1] School of Food Science and Nutrition, University of Leeds, Leeds LS2 9JT, UK; o.o.adekolurejo@leeds.ac.uk (O.O.A.); a.r.mackie@leeds.ac.uk (A.R.M.)
[2] Faculty of Biological Sciences, University of Leeds, Leeds LS2 9JT, UK; k.mcdermott@leeds.ac.uk (K.M.); h.m.r.greathead@leeds.ac.uk (H.M.R.G.); h.m.miller@leeds.ac.uk (H.M.M.)
* Correspondence: c.bosch@leeds.ac.uk

Table S1. Growth performance and feed intake of weaned pigs

Parameters	CON	ZNO	RB2	RB4	SEM	*P* value
Initial BW (kg)	7.90	7.64	7.69	8.02	0.186	0.883
Final BW (kg)	9.11	8.67	9.37	9.59	0.259	0.640
Weight gain (kg)	1.21	1.04	1.67	1.57	0.214	0.700
ADFI (kg/day)	0.18	0.17	0.25	0.26	0.016	0.104
ADG (kg/day)	0.09	0.07	0.12	0.11	0.016	0.684
FCR	2.14	2.31	2.11	2.29	0.089	0.747
*Fecal score						
Day 0	2.5	3.5	2.0	2.0	0.354	0.108
Day 7	2.5	2.0	2.5	2.5	0.125	0.804
Day 14	2.0	2.0	2.0	2.0	0.000	1.000

Data presented as mean with standard error of mean (SEM), CON, ZNO, RB2 and RB4 represents; control diet, diet supplemented with zinc oxide, 2% and 4% red beetroot respectively. ADFI; Average daily feed intake, ADG; Average daily gain, FCR; Feed conversion ratio.
*where 1; firm feces, 2; soft feces, 3; mild diarrhoea, 4; severe diarrhoea and 5; scour.

Table S2. Comparison of mean relative abundance of top bacteria genera between diet groups and gut location

Genera	Diets				Gut locations			[d]SEM	*P* value		
	CON	ZNO	RB2	RB4	Jejunum	Ileum	Caecum		[e]D	[f]L	[g]L* D
Megasphaera	35.21^{a}	15.90^{b}	12.49^{b}	24.95^{a}	18.18^{b}	14.57^{b}	33.66^{a}	2.78	0.011	0.007	0.441
Streptococcus	22.43^{b}	33.72^{ab}	44.77^{a}	38.81^{a}	47.03^{a}	48.97^{a}	8.79^{b}	2.95	0.004	0.000	0.661
Lactobacillus	6.30^{a}	4.36^{a}	3.73^{a}	3.17^{a}	4.57^{a}	5.40^{a}	3.20^{a}	0.48	0.104	0.161	0.594
Lactococcus	5.20^{a}	4.04^{a}	5.68^{a}	3.03^{a}	8.53^{a}	4.84^{b}	0.9^{c}	0.75	0.511	0.000	0.605
Lactobacillales_unclassified	4.55^{b}	7.62^{a}	6.08^{a}	6.75^{a}	7.14^{a}	9.27^{a}	2.33^{b}	0.58	0.166	0.000	0.376
Mitsuokella	3.51^{a}	2.23^{a}	1.38^{a}	2.49^{a}	3.35^{a}	2.54^{a}	1.31^{a}	0.53	0.575	0.303	0.846
Veillonellaceae_unclassified	2.73^{a}	0.12^{b}	0.07^{b}	0.25^{b}	0.94^{a}	0.96^{a}	0.49^{a}	0.30	0.003	0.750	0.895
Selenomonadaceae_unclassified	1.95^{a}	0.12^{a}	1.19^{a}	0.35^{a}	0.36^{b}	0.24^{b}	2.11^{a}	0.32	0.139	0.024	0.518
Olsenella	1.89^{a}	0.68^{ab}	0.24^{b}	0.36^{b}	0.82^{a}	1.03^{a}	0.53^{a}	0.18	0.004	0.494	0.171
Bifidobacterium	1.63^{a}	0.46^{ab}	0.12^{b}	0.60^{ab}	0.60^{a}	0.99^{a}	0.52^{a}	0.17	0.011	0.468	0.778
Phascolarctobacterium	1.42^{a}	3.75^{a}	3.98^{a}	3.06^{a}	0.03^{b}	0.04^{b}	9.09^{a}	0.66	0.245	0.000	0.222
Streptococcaceae_unclassified	1.36^{a}	2.14^{a}	1.75^{a}	1.58^{a}	2.94^{a}	1.97^{b}	0.22^{c}	0.19	0.322	0.000	0.829
Firmicutes_unclassified	1.08^{a}	0.83^{a}	0.92^{a}	0.67^{a}	0.30^{b}	0.46^{b}	1.86^{a}	0.10	0.238	0.000	0.004
Bacteria_unclassified	0.95^{a}	1.37^{a}	1.13^{a}	1.26^{a}	0.17^{b}	0.28^{b}	3.09^{a}	0.21	0.796	0.000	0.800
Erysipelotrichaceae_unclassified	0.64^{ab}	1.35^{a}	0.23^{b}	0.15^{b}	0.29^{b}	0.36^{ab}	1.12^{a}	0.15	0.006	0.018	0.001
Negativibacillus	0.44^{b}	1.91^{a}	1.29^{ab}	0.30^{b}	0.009^{b}	0.004^{b}	2.94^{a}	0.29	0.075	0.000	0.038
Anaerovibrio	0.30^{b}	0.26^{b}	1.09^{a}	2.03^{a}	0.027^{b}	0.014^{b}	2.72^{a}	0.29	0.036	0.000	0.012
Bacilli_unclassified	0.29^{b}	0.64^{ab}	0.99^{a}	0.67^{ab}	0.68^{ab}	0.89^{a}	0.38^{b}	0.07	0.000	0.001	0.003
Rumminococcaceae_unclassified	0.29^{b}	0.97^{a}	0.43^{ab}	0.23^{b}	0.01^{b}	0.01^{b}	1.42^{a}	0.12	0.013	0.000	0.002
Terrisporobacter	0.19^{c}	3.41^{a}	2.16^{ab}	1.10^{bc}	0.48^{b}	1.66^{ab}	3.00^{a}	0.31	0.000	0.001	0.015
Clostridiaceae_unclassified	0.17^{b}	2.15^{a}	1.50^{ab}	1.14^{ab}	0.16^{b}	1.97^{a}	1.59^{a}	0.24	0.013	0.002	0.111

Values represents mean abundance genera for each diet and gut location, significant differences indicated by different letters within diet groups and gut location. ANOVA and Tukey's HSD test was used to check for significant differences between the means. CON, ZNO, RB2 and RB4 represents control diet, diets supplemented with zinc oxide, 2% and 4% red beetroot respectively. [d] overall mean for each phylum, [d] Standard error of mean, [e] *p* value for significant effect of diets, [f] *p* value for significant effect of gut locations, [g] *p* value interaction between gut location and diets.

(**a**) Jejunum

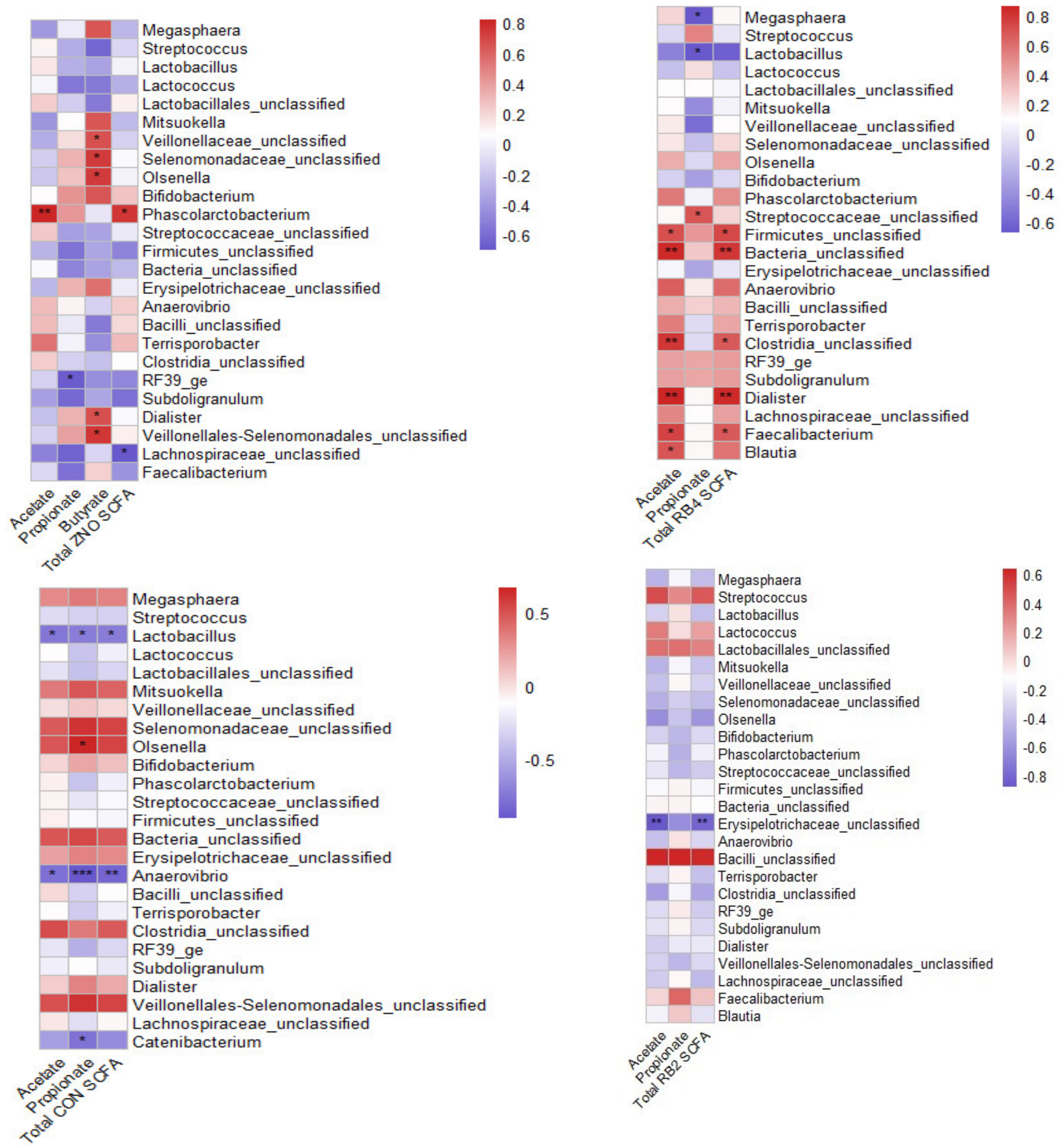
Megasphaera
Streptococcus
Lactobacillus
Lactococcus
Lactobacillales_unclassified
Mitsuokella
Veillonellaceae_unclassified
Selenomonadaceae_unclassified
Olsenella
Bifidobacterium
Phascolarctobacterium
Streptococcaceae_unclassified
Firmicutes_unclassified
Bacteria_unclassified
Erysipelotrichaceae_unclassified
Anaerovibrio
Bacilli_unclassified
Terrisporobacter
Clostridia_unclassified
RF39_ge
Subdoligranulum
Dialister
Veillonellales-Selenomonadales_unclassified
Lachnospiraceae_unclassified
Faecalibacterium
Acetate
Propionate
Butyrate
Total ZNO SCFA
0.8
0.6
0.4
0.2
0
-0.2
-0.4
-0.6
Megasphaera
Streptococcus
Lactobacillus
Lactococcus
Lactobacillales_unclassified
Mitsuokella
Veillonellaceae_unclassified
Selenomonadaceae_unclassified
Olsenella
Bifidobacterium
Phascolarctobacterium
Streptococcaceae_unclassified
Firmicutes_unclassified
Bacteria_unclassified
Erysipelotrichaceae_unclassified
Anaerovibrio
Bacilli_unclassified
Terrisporobacter
Clostridia_unclassified
RF39_ge
Subdoligranulum
Dialister
Lachnospiraceae_unclassified
Faecalibacterium
Blautia
Acetate
Propionate
Total RB4 SCFA
0.8
0.6
0.4
0.2
0
-0.2
-0.4
-0.6
Megasphaera
Streptococcus
Lactobacillus
Lactococcus
Lactobacillales_unclassified
Mitsuokella
Veillonellaceae_unclassified
Selenomonadaceae_unclassified
Olsenella
Bifidobacterium
Phascolarctobacterium
Streptococcaceae_unclassified
Firmicutes_unclassified
Bacteria_unclassified
Erysipelotrichaceae_unclassified
Anaerovibrio
Bacilli_unclassified
Terrisporobacter
Clostridia_unclassified
RF39_ge
Subdoligranulum
Dialister
Veillonellales-Selenomonadales_unclassified
Lachnospiraceae_unclassified
Catenibacterium
Acetate
Propionate
Total CON SCFA
0.5
0
-0.5
Megasphaera
Streptococcus
Lactobacillus
Lactococcus
Lactobacillales_unclassified
Mitsuokella
Veillonellaceae_unclassified
Selenomonadaceae_unclassified
Olsenella
Bifidobacterium
Phascolarctobacterium
Streptococcaceae_unclassified
Firmicutes_unclassified
Bacteria_unclassified
Erysipelotrichaceae_unclassified
Anaerovibrio
Bacilli_unclassified
Terrisporobacter
Clostridia_unclassified
RF39_ge
Subdoligranulum
Dialister
Veillonellales-Selenomonadales_unclassified
Lachnospiraceae_unclassified
Faecalibacterium
Blautia
Acetate
Propionate
Total RB2 SCFA
0.6
0.4
0.2
0
-0.2
-0.4
-0.6
-0.8

(**b**) Ileum

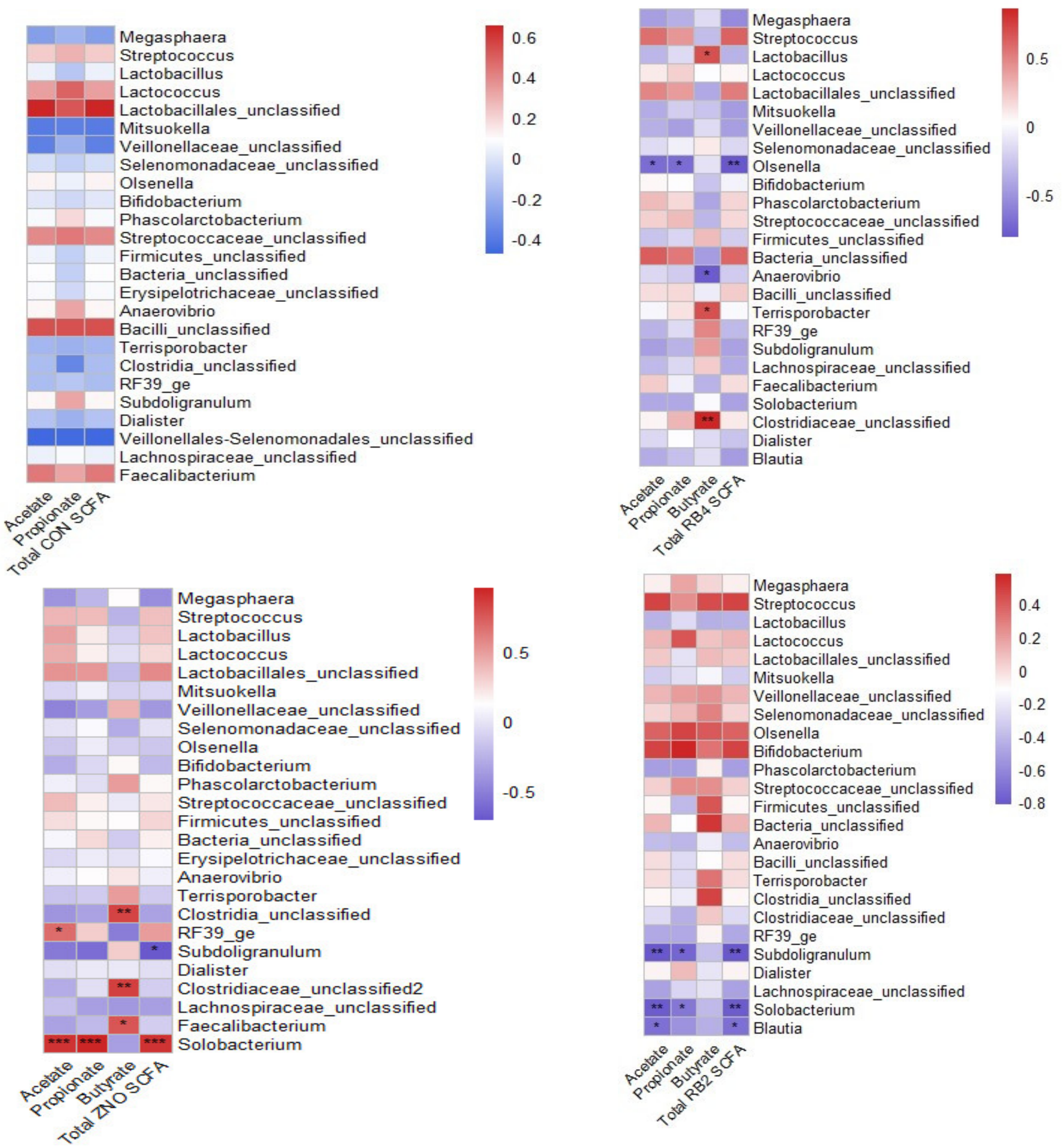

Figure S1. Spearman correlation matrices between top abundant bacteria genera and short chain fatty acids in the (**a**) jejunum(**b**) ileum. Omitted fatty acids were not detected in the corresponding location hence not shown, color depth depicts correlation between genera and gut metabolite where red color denotes a positive correlation and blue color a negative correlation. The strength of association between the subjects is indicated by the color intensity, *** $P \leq 0.001$, **$P \leq 0.01$, *$P \leq 0.05$). CON, ZNO, RB2 and RB4 represent control diet and diets supplemented with zinc oxide, 2% and 4% red beetroot respectively.

(**a**) Ileum

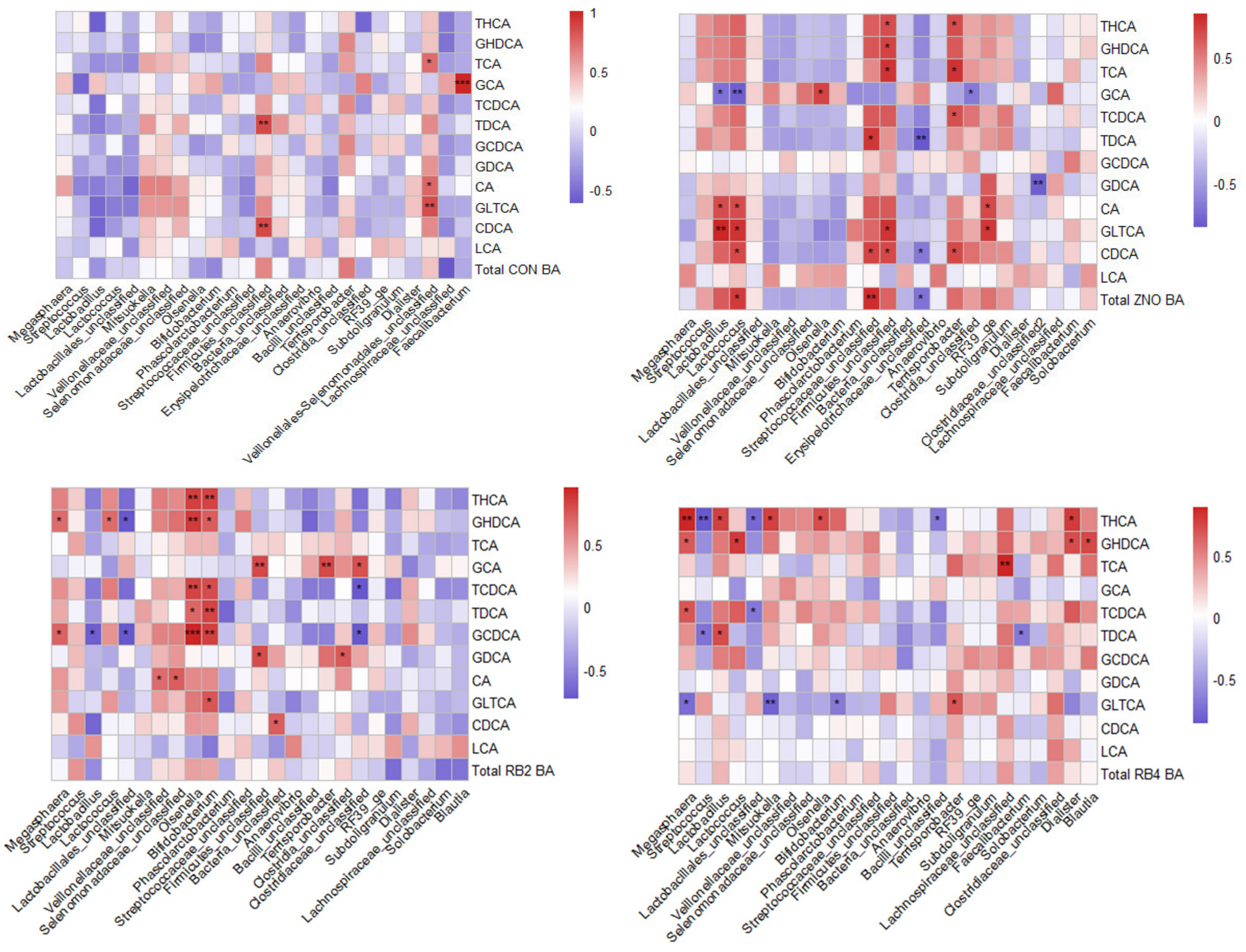
THCA
GHDCA
TCA
GCA
TCDCA
TDCA
GCDCA
GDCA
CA
GLTCA
CDCA
LCA
Total CON BA
1
0.5
0
-0.5
Megasphaera
Streptococcus
Lactobacillus
Lactococcus
Lactobacillales_unclassified
Mitsuokella
Veillonellaceae_unclassified
Selenomonadaceae_unclassified
Olsenella
Bifidobacterium
Phascolarctobacterium
Streptococcaceae_unclassified
Firmicutes_unclassified
Bacteria_unclassified
Erysipelotrichaceae_unclassified
Anaerovibrio
Bacilli_unclassified
Terrisporobacter
Clostridia_unclassified
RF39_ge
Subdoligranulum
Dialister
Veillonellales-Selenomonadales_unclassified
Lachnospiraceae_unclassified
Faecalibacterium
Total ZNO BA
Clostridiaceae_unclassified2
Solobacterium
Total RB2 BA
Clostridiaceae_unclassified
Blautia
Total RB4 BA

(**b**) Caecum

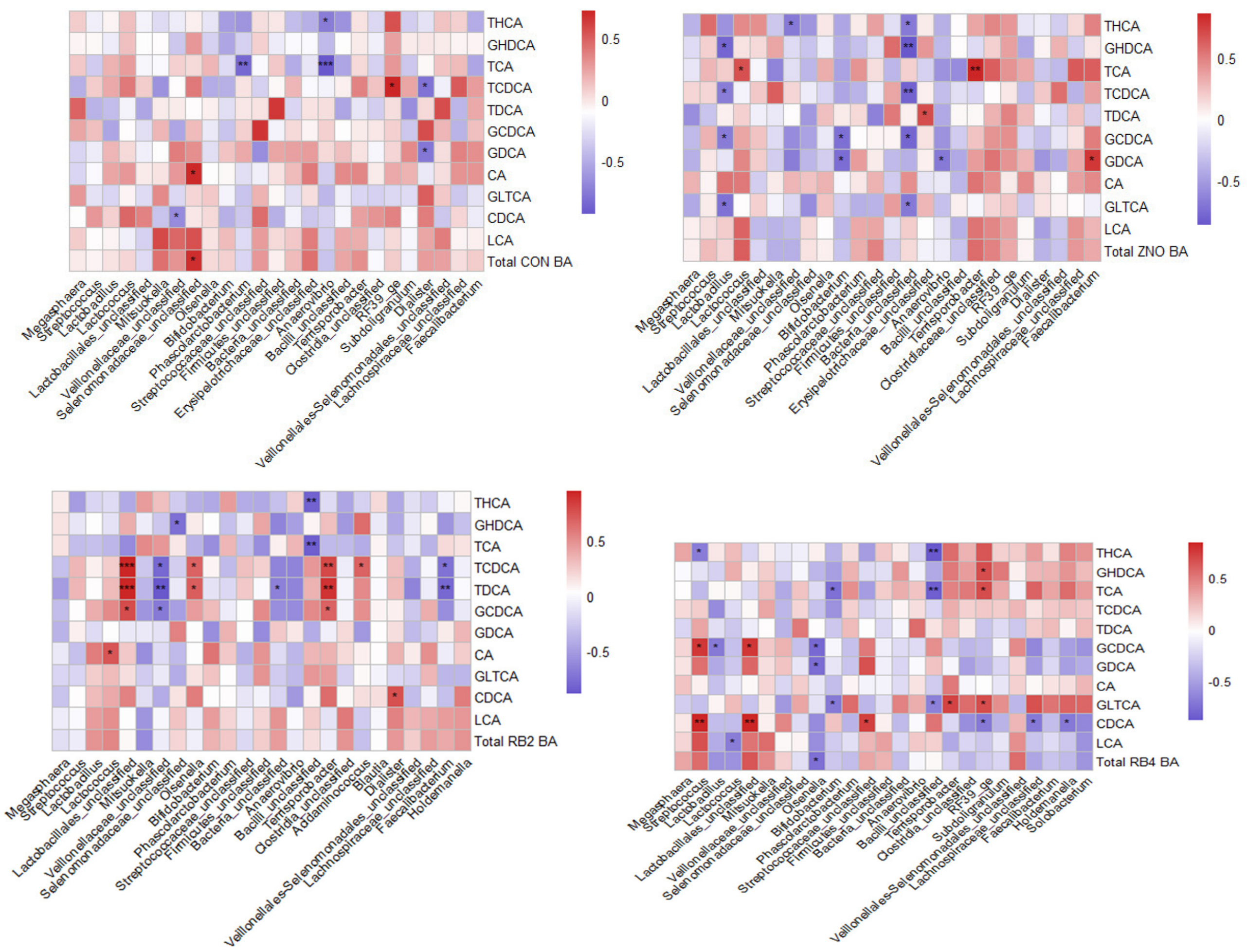

Figure S2. Spearman correlation matrices between top abundant bacteria genera and bile acid levels in the (**a**) ileum (**b**) caecum. Omitted bile acid (deoxycholic acid - DCA) was not detected in the jejunum for the pigs hence not shown. Correlation depicted by color depth, where red denotes a positive and blue a negative correlation. The strength of association between the subjects is indicated by the color intensity and *** $P \leq 0.001$, **$P \leq 0.01$, *$P \leq 0.05$). CON, ZNO, RB2 and RB4 represent control and diets supplemented with zinc oxide, 2% and 4% red beetroot respectively. Taurohyodeoxycholic acid (THCA), glycohyodeoxycholic acid (GHDCA), taurocholic acid (TCA), glycocholic acid (GCA), taurochenodeoxycholic acid (TCDCA), taurodeoxycholic acid (TDCA), glycochenodeoxycholic acid (GCDCA), glycodeoxycholic acid (GDCA), cholic acid (CA), glycolithocholic acid (GLTHCA), chenodeoxycholic.
